# Duration of clopidogrel treatment and risk of mortality and recurrent myocardial infarction among 11 680 patients with myocardial infarction treated with percutaneous coronary intervention: a cohort study

**DOI:** 10.1186/1471-2261-10-6

**Published:** 2010-01-29

**Authors:** Rikke Sørensen, Steen Z Abildstrom, Peter Weeke, Emil L Fosbøl, Fredrik Folke, Morten L Hansen, Peter R Hansen, Jan K Madsen, Ulrik Abildgaard, Lars Køber, Henrik E Poulsen, Christian Torp-Pedersen, Gunnar H Gislason

**Affiliations:** 1 Department of Cardiology, Copenhagen University Hospital Gentofte, Niels Andersens Vej 65, 2900 Hellerup, Denmark; 2 Cardiovascular Research Unit, Department of Internal Medicine, Copenhagen University Hospital Glostrup, Ndr. Ringvej 57, 2600 Glostrup, Denmark; 3 National Institute of Public Health, University of Southern Denmark, Øster Farimagsgade 5A, second floor, 1353 København K, Denmark; 4 The Heart Centre, Copenhagen University Hospital Rigshospitalet, Blegdamsvej 9, 2100 København Ø, Denmark; 5 Department of Clinical Pharmacology, Copenhagen University Hospital Bispebjerg, Bispebjerg Bakke 23, 2400 København NV, Denmark; 6 Laboratory of Clinical Pharmacology, Q7642, Copenhagen University Hospital Rigshospitalet, Blegdamsvej 9, 2100 København Ø, Denmark

## Abstract

**Background:**

The optimal duration of clopidogrel treatment after percutaneous coronary intervention (PCI) is unclear. We studied the risk of death or recurrent myocardial infarction (MI) in relation to 6- and 12-months clopidogrel treatment among MI patients treated with PCI.

**Methods:**

Using nationwide registers of hospitalizations and drug dispensing from pharmacies we identified 11 680 patients admitted with MI, treated with PCI and clopidogrel. Clopidogrel treatment was categorized in a 6-months and a 12-months regimen. Rates of death, recurrent MI or a combination of both were analyzed by the Kaplan Meier method and Cox proportional hazards models. Bleedings were compared between treatment regimens.

**Results:**

The Kaplan Meier analysis indicated no benefit of the 12-months regimen compared with the 6-months in all endpoints. The Cox proportional hazards analysis confirmed these findings with hazard ratios for the 12-months regimen (the 6-months regimen used as reference) for the composite endpoint of 1.01 (confidence intervals 0.81-1.26) and 1.24 (confidence intervals 0.95-1.62) for Day 0-179 and Day 180-540 after discharge. Bleedings occurred in 3.5% and 4.1% of the patients in the 6-months and 12-months regimen (p = 0.06).

**Conclusions:**

We found comparable rates of death and recurrent MI in patients treated with 6- and 12-months' clopidogrel. The potential benefit of prolonged clopidogrel treatment in a real-life setting remains uncertain.

## Background

Clopidogrel reduces coronary ischemic events in patients with acute coronary syndrome[1] and after percutaneous coronary intervention (PCI) where the beneficial effect is evident within the first 24 hours of treatment initiation[2-4]. In the past 5 years there has been a clear tendency to recommend increased duration of clopidogrel treatment. Current guidelines recommend 12 months of treatment for all patients after non-ST-elevation myocardial infarction, ST-elevation myocardial infarction and after treatment with PCI, in the absence of a high risk of bleeding[5-7]. The optimal duration of clopidogrel treatment is a major clinical issue, as clopidogrel, in addition to the desired anti-thrombotic effect, poses a considerable risk of bleeding. Premature cessation has been associated with increased risk of thrombotic events, including stent thrombosis[8,9]. No randomized study has addressed the optimal length of clopidogrel treatment, and current guidelines rely on the arbitrary choice of approximately 12 months' treatment used in prior studies[1-3]. Notably, the major benefit of clopidogrel treatment was observed during the first months of treatment in these studies[1-4]. The benefit of clopidogrel may decline with time, but the risk of bleeding remains; therefore, further studies of the optimal length of treatment are warranted.

In Denmark, the length of clopidogrel treatment has followed the change in guidelines, increasing from 6 months in 2002-2003 to 12 months after 2004[10]. We conducted a nationwide study of 11680 PCI treated patients with acute myocardial infarction (MI) to evaluate additional benefit (death or recurrent MI) and safety (bleedings) from the increased duration of clopidogrel treatment.

## Methods

Denmark has several nationwide registers that enable surveillance of the Danish population over time. By use of a personal civil registration number, detailed information from different registries can be linked on an individual level. The Danish National Patient Register holds information on all admissions to Danish hospitals since 1978. Every admission is registered by diagnoses according to the International Classification of Diseases. The Danish Register of Medicinal Product Statistics contains information on all prescriptions dispensed in Danish pharmacies since 1995, with information of Anatomic Therapeutic Chemical (ATC) code, strength, and number of tablets dispensed. Due to partial reimbursement of drug expenses by the national government-financed health care system, pharmacies are required to register all dispensed prescriptions, which ensures complete registration nationwide[11]. In Denmark, most cardiovascular pharmaceuticals require a prescription; however, aspirin is also dispensed over-the-counter. Patients in chronic aspirin treatment usually receive aspirin on prescription in order to receive financial reimbursement. Information of vital status of all citizens is held in the Civil Register.

### Population

We used the Danish National Patient Register to identify all patients admitted with first-time MI, classified as International Classification of Diseases-10 codes I21 and I22, to Danish hospitals between 2002 and 2005. Subsequent PCI was identified by the Danish health care classification system codes KFNG02 and KFNG05. Our study population consisted of MI patients treated with clopidogrel and PCI within 30 days after date of admission. The diagnosis of MI has previously been validated to have a specificity of 93%[12]. However, the register cannot accurately distinguish between the diagnoses non-ST-elevation myocardial infarction and ST-elevation myocardial infarction. To compensate for this, we classified the patients into groups according to the time from admission to PCI treatment; patients treated with PCI on Day 0-1 after admission and patients treated with PCI on Day 2-29. Of the patients treated with PCI on Day 0-1 57.3% were coded as transmural MI (I21.0 -I21.3), 9.2% as subendocardial MI (I21.4), 33.1% with unspecified MI (I21.9) and 0.4% with other types (I22.0-I22.9). Corresponding figures for patients treated with PCI on Day 2-29 were 12.5%, 55.3%, 31.7% and 0.5%. Co-morbidity was determined according to the modified Ontario Acute Myocardial Infarction Mortality Prediction Rules by diagnosis from the index admission and one year before admission[13]. The diagnosis of heart failure has a low sensitivity in the National Patient Register[14]. Therefore, claimed prescriptions of loop diuretics 90 days before admission until 90 days after discharge were used as a proxy for heart failure, a method used previously[15]. Similarly, diabetes was considered present in patients claiming a prescription for glucose-lowering drugs (ATC A10). The following International Classification of Diseases-10 codes were used for the safety endpoint (bleedings): I60-I62, S06.4-06.6, J94.2, R04, R31, K25.0, K25.2, K25.4, K26.0, K26.2, K26.4, K27.0, K27.2, K28.0, K28.2, K92.0-92.2, D62, D50.

### Clopidogrel treatment and concomitant cardiovascular pharmacotherapy

**Figure 1:**
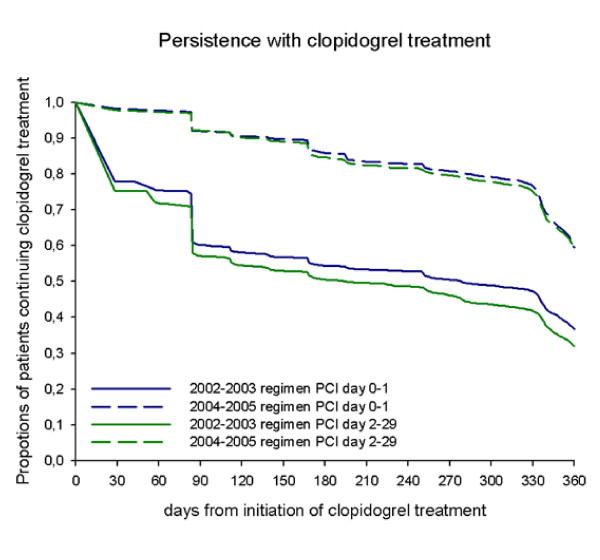
**Persistence with clopidogrel treatment**.

Use of clopidogrel was defined as a dispensed prescription within 30 days of discharge (ATC code B01AC04). Mean length of treatment was 172.8 days (standard deviation 133.2) in 2002-2003 and 319.7 days (standard deviation 72.0) in 2004-2005, calculated from the proportions of days covered[10]. Subdivision in treatment periods, with corresponding variance of treatment duration of 6-months and 12-months treatment, respectively, is referred to as the 2002-2003 regimen and the 2004-2005 regimen. The persistence with treatment is shown in Figure 1. Information of coverage of clopidogrel tablets on Day 91 and Day 181 after first dispensed prescription of clopidogrel were calculated and used in a sensitivity analysis.

Concomitant pharmacotherapy with the following drugs was defined as a prescription dispensed within 90 days of discharge: beta-blockers (ATC code C07), angiotensin-converting enzyme inhibitors and angiotensin-II receptor blockers (ATC code C09), spironolacton (ATC code C03DA), or vitamin K antagonists (ATC code B01AA). Statins (ATC code C10AA) were registered as a prescription dispensed within 180 days after admission, and aspirin treatment (ATC code B01AC06, N02BA01) was identified by dispensed prescriptions 90 days before until 90 days after admission.

### Outcomes

We studied the effect of clopidogrel treatment in relation to the following endpoints: 1. all-cause mortality; 2. recurrent MI, defined as a re-admission to a Danish hospital with the diagnosis of MI more than 30 days after discharge from date of admission for MI; and 3. combined endpoint of death or recurrent MI. Individuals were followed for 18 months and censored at the time of the first event. Patients included after July 1, 2005 were followed until December 31, 2006. A safety endpoint was used to quantify the proportion of patients experiencing a major bleeding, defined as an admission to a hospital with a bleeding diagnosis or death caused by bleeding. The bleedings were assessed up to 18 months of follow-up, individuals were censored at first bleeding event.

### Statistical Analysis

Descriptive statistics and baseline variables are presented as percentages or means with standard deviation. Chi^2 ^test was used to assess differences between categorical data; the Student's t-test was used for continuous data. For survival analysis we performed landmark-analyses using the Kaplan-Meier method (until 18 months of follow-up), the first analysis covered Day 0-179 and the second started on Day 180 after date of admission for MI, and included patients alive and without events at this time point. The time of the landmark analysis was chosen because the mean duration of treatment in the 2002-2003 regimen was172.8 days (standard deviation 133.2) and was performed to illustrate the potential gain from the extended clopidogrel treatment in the 2004-2005 regimen (mean treatment 319.7 days, standard deviation 72.0)[10]. Adjusted event rates were estimated by Cox proportional hazards models, using the 2002-2003 regimen as reference, adjusted for the following variables: age, sex, concomitant cardiovascular treatment (use of β-blockers, angiotensin-converting enzyme inhibitors, angiotensin II receptor blockers, statins, loop-diuretics, glucose-lowering drugs, vitamin K antagonists, aspirin), and co-morbidity (cerebral vascular disease, cardiac dysrythmias, diabetes with complications, acute renal failure, chronics renal failure, malignancy, shock , pulmonary odema). To match the population of the landmark analyses, the 2004-2005 regimen was split into two time-dependent variables: Clopidogrel treatment Day 0-180 and Clopidogrel treatment Day 180-540. Patients treated with PCI on Day 0-1 and Day 2-29 were analysed separately. The proportional hazard-assumption, linearity of continuous variables and lack of interactions, were found to be valid. Due to less favourable persistence with clopidogrel treatment in the 2002-2003 regimen, a sensitivity analysis was performed. The sensitivity landmark analysis compared the endpoint among patients with or without clopidogrel treatment at Day 91 and Day 181. Corresponding Cox analyses of these patients were made, with no clopidogrel treatment used as reference.

All statistical calculations were performed using the SAS statistical software package, version 9.1 for Windows (SAS Institute Inc., Cary, NC, USA).

## Ethics

Retrospective register studies do not require ethical approval in Denmark. The study was approved by the Danish Data Protection Agency (ref: 2003-54-1269).

## Results

### Baseline characteristics

**Figure 2:**
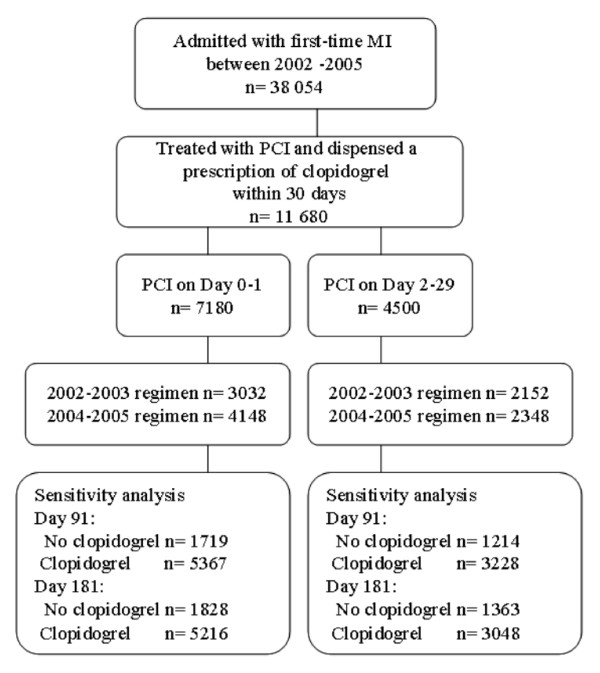
**The study population**.

Between 2002 and 2005, a total of 11680 patients were hospitalised with a first-time diagnosis of MI, were treated with PCI within 30 days and dispensed a prescription of clopidogrel within 30 days after discharge (Figure 2). Baseline characteristics are shown in Additional file 1.

### Landmark analyses: effect of different duration of clopidogrel treatment

**Figure 3:**
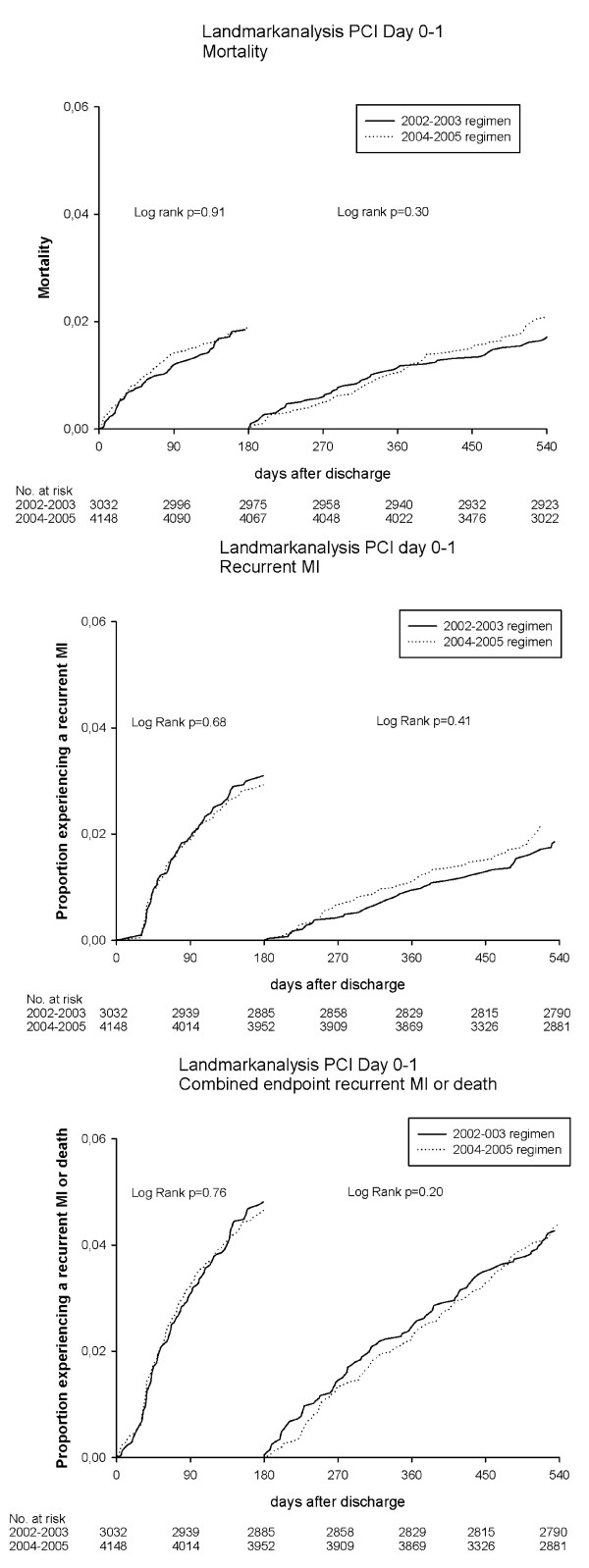
**Landmark analyses using the Kaplan Meier Methods for patients with PCI on Day 0-1**. Patients event-free on Day 180 were included in the second analysis.

**Figure 4:**
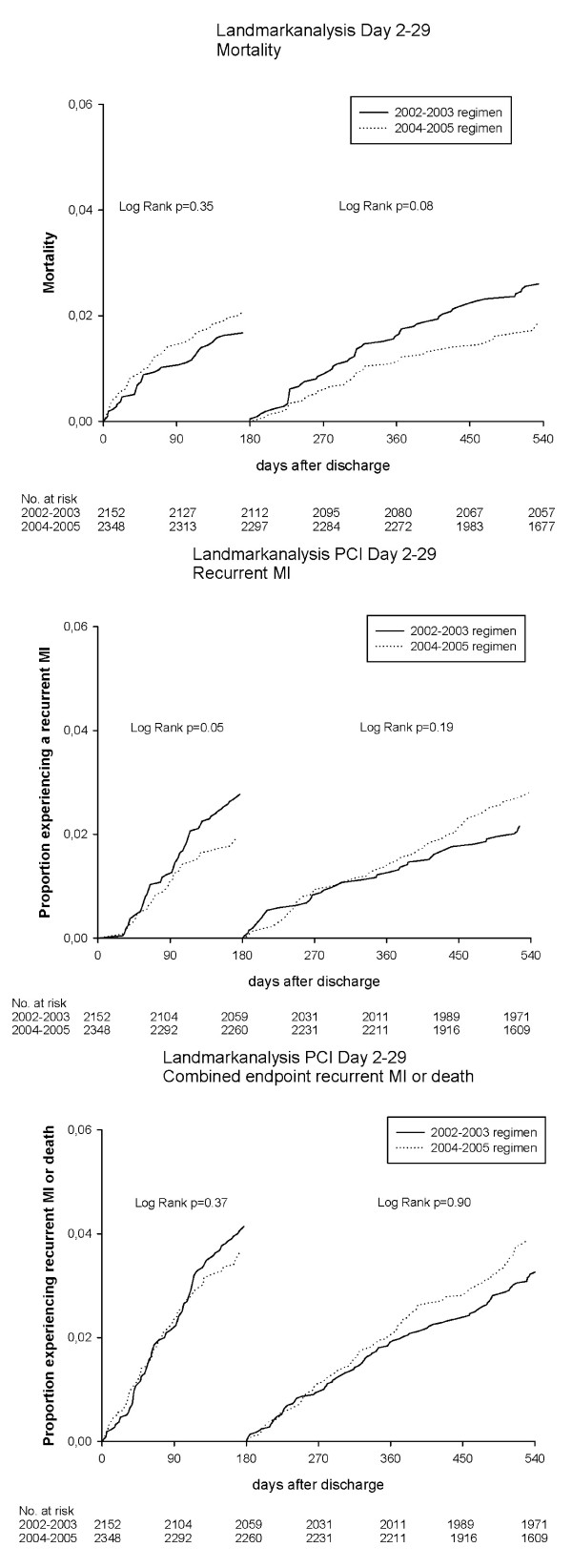
**Landmark analyses using the Kaplan Meier Methods for patients with PCI on day 2-29**. Patients event-free on Day 180 were included in the second analysis.

Results of the landmark analyses are shown in Figure 3 and 4. The landmark analyses showed no differences in death, recurrent MI or the combined endpoint, when comparing the 2002-2003 regimen with the 2004-2005 regimen among patients treated with PCI Day 0-1. Likewise, the landmark analyses among patients treated invasively on Day 2-29 showed no differences in rates of death or combined endpoint. For recurrent MI there was a reduced occurrence among patients treated during 2004-2005 within the first 180 days of treatment (p = 0.05).

### Cox proportional hazard analyses

**Figure 5:**
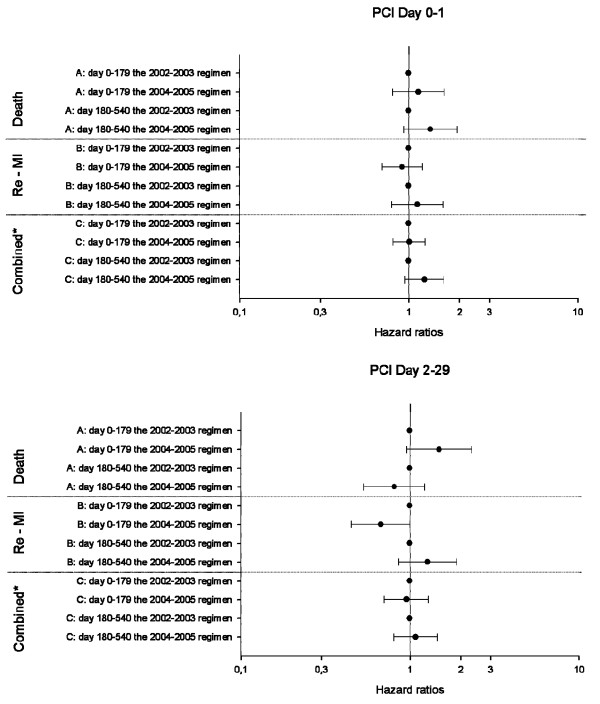
Results of Cox proportional hazards Model*: 5A illustrates patients treated with PCI on Day 0-1 and 5B patients treated on Day 2-29. * The models were adjusted for age, sex, co-morbidities (cerebral vascular disease, diabetes with complications, cardiac dysrythmias, acute renal disease, chronic renal disease, malignancy, shock, pulmonary odema), concomitant medications (β-blockers, angiotensin-converting enzyme inhibitors and angiotensin-II receptor blockers, statins, loop diuretics, antidiabetic medication, vitamin K antagonists, aspirin).

The Cox proportional hazards models showed no difference between the 2002-2003 regimen and the 2004-2005 regimen in all endpoints (Figure 5). This applied to both patients with PCI Day 0-1 and Day 2-29.

### Safety

We found a total of 440 (3.8%) hospitalizations for bleedings or deaths caused by bleeding within 18 months after discharge, evenly distributed between patients treated with PCI on Day 0-1 and Day 2-29. In the 2002-2003 regimen, 176 (3.5%) patients had bleedings, corresponding to 264 (4.1%) patients in the 2004-2005 regimen (p = 0.06).

### Sensitivity analyses

Among patients treated with PCI Day 0-1, a total of 1719 were without clopidogrel treatment Day 91 and 1828 without clopidogrel treatment Day181. Of these 73.7% and 71.1% were admitted during 2002-2003, respectively. Among patients treated with PCI day 2-29, 1214 and 1363 were without clopidogrel treatment Day 91 and Day 181 (79.6% and 74.9% were admitted during 2002-2003). Results of the sensitivity landmark analyses and the sensitivity Cox proportional hazards model are shown in Table 1.

**Table 1:** Sensitivity analysis

	PCI Day 0-1		PCI Day 2-29	
Landmark analyses	Log rank	P-value	Log rank	P-value
**Day 91-180**				
Death		0.11		0.002
Recurrent MI		0.17		0.07
Combined		0.06		0.007
**Day 181-540**				
Death		0.25		0.001
Recurrent MI		0.33		0.99
Combined		0.65		0.1
**Cox Proportional Hazards Model**	HR (CI)*	**P-value**	HR (CI)*	**P-value**

**Day 91-180**				
**Death**				
No clopidogrel	1.00		1.00	
Clopidogrel	0.49 (0.23-0.97)	0.04	0.28 (0.12-0.64)	0.003
**Recurrent MI**				
No clopidogrel	1.00		1.00	
Clopidogrel	0.71 (0.42-1.19)	0.19	0.75 (0.40-1.38)	0.35
**Combined**				
No clopidogrel	1.00		1.00	
Clopidogrel	0.66 (0.43-1.02)	0.06	0.62 (0.37-1.04)	0.07
**Day 181-540**				
**Death**				
No clopidogrel	1.00		1.00	
Clopidogrel	0.83 (0.55-1.23)	0.34	0.55 (0.35-0.86)	0.01
**Recurrent MI**				
No clopidogrel	1.00		1.00	
Clopidogrel	1.17 (0.76-1.81)	0.48	0.79 (0.50-1.24)	0.3
**Combined**				
No clopidogrel	1.00		1.00	
Clopidogrel	0.92 (0.68-1.25)	0.58	0.73 (0.53-1.02)	0.07

* Hazard ratio (confidence intervals).

## Discussion

This nationwide study of PCI treated MI patients examined the difference in effect and safety of 6- and 12-months clopidogrel treatment. The main result is that clopidogrel treatment beyond 6 months provided no benefit in rates of death, recurrent MI or a combined endpoint. We found a trend of increased number of bleedings among patients treated in the 2004-2005 regimen compared with those treated with the 2002-2003 regimen (p = 0.06).

Several studies have addressed the question of different durations of clopidogrel treatment after acute coronary syndrome and PCI[2,3,16,17]. Eisenstein et al reported benefit of 12 months treatment compared with 6 months in patients treated with drug-eluting stents and concluded that at least 12 months' treatment was appropriate in this group of patients with lifelong treatment as a possibility. Patients treated with bare-metal stents had no advantage in respect to recurrent MI or death with clopidogrel treatment >6 months[16]. The main weakness of the study by Eisenstein et al is that the choice of 6 or 12 months' treatment for the patient was based on a decision by the patient or the physician and was thus subject to confounding by indication. In comparison, our study avoided this selection of patients as we compared different treatment regimens based on standardized recommendations. The randomized Clopidogrel for the Reduction of Events During Observation (CREDO) trial found that 12 months' clopidogrel treatment was superior to 2-4 weeks of treatment[3]. The PCI-CURE trial revealed a superior effect of combined treatment with aspirin and clopidogrel vs. aspirin in non-ST-elevation MI/acute coronary syndrome patients treated with PCI. The main benefit was observed from Day 2 to Day 30. Considering the period from PCI to end of follow-up, the PCI-CURE investigators found less occurrence of MI in the clopidogrel + aspirin group but comparable mortality-rates between the groups. However, the trial did not report the additional reduction of events that happened from Day 30 after PCI to end of follow-up, as no landmark analysis was presented. In the PCI-CURE study all stent-treated PCI patients received 2-4 weeks open-label clopidogrel followed by the randomized study drug[2]. Eisenstein et al, the CREDO study and the PCI-CURE study did not report differences in event rates between 6- and 12-months' treatment, hence a direct comparison with our study is not possible.

In our sensitivity analysis, where persistence with treatment was ensured, we found no additional effect of 12 months treatment compared with 6 months treatment among patients with PCI Day 0-1. In patients treated with PCI Day 2-29, a reduced mortality was found among patients in clopidogrel treatment more than 6 months (Log rank p = 0.001, HR 0.55 (CI 0.35-0.86), p-value 0.01). Thus, the sensitivity analyses confirm the findings from the main analysis, except in patients treated with PCI day 2-29, where we found an effect on mortality. However, this result should be interpreted with caution, since it could be due to selection bias of low risk patients (with no endpoint the first six month of the study).

Other studies have focused on variations in clinical outcome between different stent-types (bare-metal stents and drug-eluting stents) and found small differences in early and late event rates[9,16,18,19]. In these studies the information on use of clopidogrel is often inadequate, and some have referred to written guidelines[18,19]. In our study we have no information on the stent types used, but another Danish study using a regional register from 2002 to mid-2005 reported the use of drug-eluting stents to be 0% in 2002 increasing to 75% in 2005[19]. We considered the effect of different durations of clopidogrel treatment on a population level in PCI treated MI patients, with regard to recurrent MI and all-cause mortality. Knowledge of the stent types and coronary lesions would have given a more detailed picture of the patients' risk profiles. The effect of prolonged clopidogrel treatment should be interpreted in the context of a substantial change of stent use in the same period and the lack of knowledge of the stent types used is a limitation of our study. An increased use of drug-eluting stents would possibly give more in-stent thromboses and thus more recurrent MIs[9,18,19]. Our results showed both unchanged rates of mortality and recurrent MIs. A meta-analysis by De Luca et al of ST-elevation MI patients treated with either bare-metal stents or drug-eluting stents compared short duration of clopidogrel treatment (3-9 months) with 12 months' treatment and found, like our study, no differences in respect to recurrent MI, death or a combination of these, between the stent types and different duration of clopidogrel treatment[17].

The risk of bleeding is increased with use of antithrombotic medication. However, there are conflicting results from both randomized and observational studies regarding bleeding risk[1-3,20]. The Clopidogrel for High Atherothrombotic Risk and Ischemic Stabilization, Management, and Advoidance (CHARISMA) trial tested long-term treatment in patients at high risk of atherothrombotic events[21]. Among the symptomatic patients, they found a significant increase of moderate bleedings[21]. The PCI-CURE and the CHARISMA study reported event rates of major bleedings of 1.6-2.7% [2,21] whereas the CREDO study had event rates of 6.7 to 8.8%[3]. Major bleedings in the CREDO trial were slightly differently defined than in the PCI-CURE and CHARISMA trial, which could explain some of the variation. In our study the average event rate for patients treated on both Day 0-1 and Day 2-29 was 3.8%. Our observed bleeding rates were higher than those reported from most randomized trials, although not as high as reported in the CREDO trial. This could be explained by the real-life setting of our study.

### Strengths and Limitations

The main strength of our study is the completeness of data, with a complete and unselected cohort of patients followed in a real-life setting. We had exact information on clopidogrel use, by individual-level linkage to dispensed prescriptions and complete data on events. Treatment duration and persistence with clopidogrel was calculated individually[10]. The concordance between drug dispensing and drug consumption is likely to be very high, since reimbursement of drug expenses is only partial in Denmark. In Denmark, aspirin can be bought as a prescribed drug as well as over-the-counter. In our study, we assessed aspirin use by dispensed prescriptions since patients in chronic treatment usually receive aspirin on prescription in order to receive financial reimbursement. This occurrence is substantiated by the high baseline use of aspirin seen in the study population (92.6%).

The main limitations of our study are inherent in the observational nature of the study. We have no information on important prognostic factors i.e. lipid levels, smoking status, or left ventricular systolic function, stent types used, coronary lesions and the treatment given during index admission, e.g. loading dose of aspirin and clopidogrel, treatment with glycoprotein IIb/IIIa inhibitors, and heparins. These factors, may have influenced the results and affected the decision by the physician to prescribe clopidogrel for a shorter or a longer period[2,22,23]. During the study period there were slight changes in the baseline characteristics of the population. However, we do not believe this had a major effect on our conclsions. More patients were treated invasively in close connection to the diagnosis of MI during the later period, with 58.5% treated Day 0-1 in 2002-2003 increasing to 63.9% in 2004-2005. This change of practice can be explained by an adaptation to national guidelines, which were altered after publication of the Danish Multicenter Randomized Study on Fibrinolytic Therapy versus Acute Coronary Angioplasty in Acute Myocardial Infarction II trial[24]. In our analyses we stratified the patients according to the time of PCI, which limited the influence on the results. We were unable to accurately distinguish between the diagnoses of non-ST-elevation myocardial infarction and ST-elevation myocardial infarction and despite we stratified the patients according to the time of PCI treatment, this is a limitation. Throughout the period a change of age and use of concomitant medical treatment were seen. We included both age and various concomitant medications in the Cox proportional hazards models to eliminate confounding by these variables, but effect of residual confounding cannot fully be excluded.

## Conclusions

This study found no beneficial effect on mortality and recurrent MI of the 12 months' clopidogrel treatment compared with 6 months' treatment in an unselected cohort of MI patients treated with PCI. Increased duration of treatment was associated with a trend toward increased occurrence of bleedings. More studies are needed to determine the potential benefits of prolonged clopidogrel treatment in a real-life setting.

## Abbreviations

(PCI): Percutaneous coronary intervention; (MI): Acute Myocardial Infarcrion; (ATC): Anatomic Therapeutic Chemical; (CREDO): Clopidogrel for the Reduction of Events During Observation trial; (CHARISMA): The Clopidogrel for High Atherothrombotic Risk and Ischemic Stabilization, Management, and Advoidance trial.

## Competing interests

The corresponding author had full access to all the data in the study and had the final responsibility for the decision to submit for publication. All authors declare that they have no competing interest.

## Authors' contributions

R.S., C.T-P, G.H.G, S.Z.A: Designed the study, and analysed data. RS obtained funding and wrote the manuscript. All authors interpreted the results, revised the paper, and approved the final version.

## Pre-publication history

The pre-publication history for this paper can be accessed here:

http://www.biomedcentral.com/1471-2261/10/6/prepub

## Supplementary Material

Additional file 1 **Baseline characteristics**. This file contains baseline information (year of admission, sex, age, co-morbidity, concomitant medical treatment). The patients are stratified according to PCI status. Click here for file
