# Supplementary material for: Duration of clopidogrel treatment and risk of mortality and recurrent myocardial infarction among 11 680 patients with myocardial infarction treated with percutaneous coronary intervention: a cohort study
Source: BMC Cardiovasc Disord. 2010 Jan 29;10:6. doi: 10.1186/1471-2261-10-6 (PMC2837608; doi:10.1186/1471-2261-10-6)
Supplement: Additional file 1 — Baseline characteristics. This file contains baseline information (year of admission, sex, age, co-morbidity, concomitant medical treatment). The patients are stratified according to PCI status. [file 1471-2261-10-6-S1.PDF]

Additional file 1. Baseline characteristics

Characteristics	All patients	PCI on Day 0–1 n=7180 Total	2002–2003 regimen	2004–2005 regimen	P-value	PCI on Day 2–29 n=4500 Total	2002–2003 regimen	2004–2005 regimen	P-value
**Total patients**	11680	7180	3032 (42.2)	4148 (57.8)		4500	2152(47.8)	2348 (52.2)	
Women	3263 (27.9)	2002	844(27.8)	1158 (27.9)	0.94	1261	632 (29.4)	629 (26.8)	0.05
Age-women*	66.5±12.1	66.2±12.6	65.7±12.6	66.7±12.7	0.08	66.8±11.3	65.6±11.3	68.0±11.1	<0.001
Men	8417 (72.1)	5178	2188(72.2)	2990 (72.0)	0.94	3239	1520 (70.6)	1719 (73.2)	0.05
Age-men*	61.3±11.5	60.7±11.9	60.2±11.8	61.1±11.8	0.009	62.2±11.0	61.7±10.8	62.6±11.1	0.03
**Co-morbidity**									
Cerebral vascular disease	203 (1.7)	113 (1.6)	43 (1.4)	70 (1.7)	0.37	90 (2.0)	45 (2.1)	45 (1.9)	0.68
Diabetes with complications	334 (2.9)	149 (2.0)	67 (2.2)	82 (2.0)	0.49	185 (4.1)	105 (4.5)	105 (4.5)	0.2
Cardiac dysrythmias	684 (5.9)	413 (5.8)	176 (5.8)	239 (5.7)	0.87	271 (6.0)	138 (5.9)	138 (5.9)	0.67
Acute renal failure	32 (0.3)	19 (0.3)	9 (0.3)	10 (0.2)	0.65	13 (0.3)	5 (0.2)	5 (0.2)	0.32
Chronic renal failure	52 (0.5)	32 (0.5)	13 (0.4)	19 (0.5)	0.85	20 (0.4)	10 (0.4)	10 (0.4)	0.85
Malignacy	139 (1.2)	88 (1.2)	31 (1.0)	57 (1.4)	0.18	51 (1.1)	24 (1.0)	24 (1.0)	0.46
Shock	56 (0.5)	45 (0.6)	19 (0.6)	26 (0.6)	0.99	11 (0.2)	2 (0.1)	2 (0.1)	0.02
Pulmonary odema	33 (0.3)	12 (0.2)	4 (0.1)	8 (0.2)	0.53	21 (0.5)	11 (0.5)	11 (0.5)	0.98
**Concomitant medical treatment**									

ß-blockers	10532 (90.2)	6538 (91.1)	2752 (90.8)	3786 (91.3)	0.46	3994 (88.8)	1898 (88.2)	2096 (89.3)	0.26
ARB**	6278 (53.8)	4179 (58.2)	1700 (56.1)	2479 (59.8)	0.002	2099 (46.6)	969 (45.0)	1130 (48.1)	0.04
Statins	11012 (94.3)	6778 (94.4)	2763 (91.1)	4015 (96.8)	<0.001	4234 (94.1)	1954 (90.8)	2280 (97.1)	<0.001
Loop diuretics†	2395 (20.5)	1471 (20.5)	594 (19.6)	877 (21.1)	0.11	924 (20.5)	450 (20.9)	474 (20.2)	0.55
Glucose-lowering drugs‡	1137 (9.7)	616 (8.6)	255 (8.4)	361 (8.7)	0.66	521 (11.6)	249 (11.6)	272 (11.6)	0.99
Vitamin K antagonists	561 (4.8)	367 (5.1)	160 (5.3)	207 (5.0)	0.59	194 (4.3)	95 (4.4)	99 (4.2)	0.74
Aspirin	10812 (92.6)	6695 (93.6)	2769 (91.3)	3926 (94.7)	<0.001	4117 (91.5)	1950 (90.6)	2167 (92.3)	0.04
Clopidogrel	11680(100.0)	7180(100.0)	3032(100.0)	4148(100.0)	.	4500(100.0)	2152(100.0)	2348(100.0)	.

Numbers in brackets are percentages.

* Mean ± SD.

**ARB=angiotensin-converting enzyme inhibitors and angiotensin-II receptor blockers

† Loop diuretic is used as a proxy for heart failure.

‡ Glucose-lowering drugs are used as a proxy for diabetes.
